# Fighting Methicillin-Resistant *Staphylococcus aureus* with Targeted Nanoparticles

**DOI:** 10.3390/ijms24109030

**Published:** 2023-05-20

**Authors:** Stéphanie Andrade, Maria J. Ramalho, Sílvio B. Santos, Luís D. R. Melo, Rita S. Santos, Nuno Guimarães, Nuno F. Azevedo, Joana A. Loureiro, Maria C. Pereira

**Affiliations:** 1 LEPABE—Laboratory for Process Engineering, Environment, Biotechnology and Energy, Faculty of Engineering, University of Porto, Rua Dr. Roberto Frias, 4200-465 Porto, Portugal; stephanie@fe.up.pt (S.A.); mjramalho@fe.up.pt (M.J.R.); ritasantos@fe.up.pt (R.S.S.); nguimaraes@fe.up.pt (N.G.); nazevedo@fe.up.pt (N.F.A.); 2 ALiCE—Associate Laboratory in Chemical Engineering, Faculty of Engineering, University of Porto, Rua Dr. Roberto Frias, 4200-465 Porto, Portugal; 3 CEB—Centre of Biological Engineering, University of Minho, 4710-057 Braga, Portugal; silviosantos@ceb.uminho.pt (S.B.S.); lmelo@deb.uminho.pt (L.D.R.M.); 4 LABBELS—Associate Laboratory, University of Minho, 4710-057 Braga, Portugal

**Keywords:** MRSA, antibiotic resistance, targeted delivery, nanoparticle functionalization, drug delivery, nanoantibiotics

## Abstract

Antimicrobial resistance (AMR) is considered one of the greatest threats to global health. Methicillin-resistant *Staphylococcus aureus* (MRSA) remains at the core of this threat, accounting for about 90% of *S. aureus* infections widespread in the community and hospital settings. In recent years, the use of nanoparticles (NPs) has emerged as a promising strategy to treat MRSA infections. NPs can act directly as antibacterial agents via antibiotic-independent activity and/or serve as drug delivery systems (DDSs), releasing loaded antibiotics. Nonetheless, directing NPs to the infection site is fundamental for effective MRSA treatment so that highly concentrated therapeutic agents are delivered to the infection site while directly reducing the toxicity to healthy human cells. This leads to decreased AMR emergence and less disturbance of the individual’s healthy microbiota. Hence, this review compiles and discusses the scientific evidence related to targeted NPs developed for MRSA treatment.

## 1. Introduction

The World Health Organization (WHO) has identified antimicrobial resistance (AMR) as one of the most serious global health problems, including this issue in the WHO’s top 10 threats faced by humanity [1]. When first discovered in 1928, antibiotics were hailed as a medical marvel, saving millions of lives worldwide. However, their misuse and overuse accelerated the emergence of drug-resistant bacteria, which are increasingly difficult or impossible to treat. Thus, bacterial infections that, in the past, were considered low-risk or easily treatable are now associated with severe morbidity and mortality [2].

Methicillin-resistant *Staphylococcus aureus* (MRSA) is the most common and one of the most dangerous antibiotic-resistant bacteria [3]. It represents the main cause of hospital-acquired infections, causing numerous diseases, such as endocarditis, chronic osteomyelitis, pneumonia, septic arthritis, osteoarthritis, and bacteremia [4]. Over the years, MRSA has developed multiple drug-resistant mechanisms to survive, including cell wall thickening, increased efflux pumps, drug target mutation, enzymatic drug modification, and biofilm formation [5]. As such, MRSA has also become resistant to a wide variety of antibiotics, including penicillin, linezolid, and daptomycin [6]. 

Due to bacterial resistance, higher doses of antibiotics are required to produce a therapeutic effect, frequently resulting in adverse effects. For instance, vancomycin, the primary drug used to treat severe MRSA infections, faces several limitations, including nephrotoxicity, low tissue penetration, and limited antibacterial activity [4]. Therefore, it is urgently necessary to develop new and effective anti-MRSA therapeutic strategies. One approach that potentially addresses these drawbacks is the use of nanoparticles (NPs) to avoid MRSA antibiotic resistance by acting either as drug delivery system (DDS) and/or as active antibacterial agents. NPs are colloidal carriers in the nanoscale (10^−9^ m) with interesting properties, including small size, large surface area, and capacity to interact with specific receptors. Their biodegradability, biocompatibility, non-immunogenicity, and high stability in body fluids are other beneficial properties of NPs that stand out [7]. Combining NPs with targeting-based strategies represents a potential approach for the delivery of high concentrations of antimicrobial agents at the infection site while decreasing the toxicity to non-target cells [8]. 

Due to the relevance of this field for the broad scientific community, some reviews focusing on the use of nanoantibiotics to fight MRSA have appeared in recent years [4,5,9,10]. The present article differs from the existing literature by presenting and discussing the development made over the years using NPs that have been designed to specifically target MRSA (in vitro) or MRSA-infected sites (in vivo), either by modifying their surface with ligands (active targeting) or by using stimuli-responsive materials (passive targeting). Thus, the present article provides the first systematic review of the NPs developed so far for the targeted therapy of MRSA. 

## 2. Properties of Nanoparticles for the Treatment of Bacterial Infections

The use of NPs seems to be an appropriate strategy to overcome AMR by acting either as DDS and/or as active antibacterial agents. When serving as drug nanocarriers, NPs can overcome bacterial resistance by protecting the loaded antibiotics from biological degradation and blocking efflux pumps [11]. Moreover, NPs allow the drug release to be controlled and sustained, thereby maintaining active doses of therapeutic agents for extended periods. Hence, a lower dose of antibacterial agent is needed to exert a therapeutic effect, thereby reducing the side effects on healthy cells/tissues [12].

In addition to serving as DDS, NPs also possess intrinsic antibacterial activity, which makes them powerful therapeutic agents for antibacterial therapy. Some types of NPs, such as metallic NPs, can produce reactive oxygen species (ROS) and reactive nitric oxide (NO) that destroy the bacterial cellular components. Moreover, NPs can inhibit DNA and enzyme synthesis, interrupt energy transduction by affecting electron transport chain reaction in the transmembrane, and release heavy metal ions with harmful effects [13]. The combination of NPs with phototherapies is another approach whereby NPs can exert antibacterial activity. Photothermal NPs absorb light and convert it into thermal energy in the surrounding medium. The consequent increase in temperature leads to chemical or physical harm to bacteria, inducing their eradication [14].

Despite their numerous advantages, directing NPs to the infection site is essential to increase the local concentration of therapeutic agents, thereby improving their antibacterial efficacy [15]. Hence, it is possible to decrease the administered dose, as well as the dosing frequency, thereby reducing toxicity to healthy tissues and overcoming drug resistance [16].

**Figure 1:**
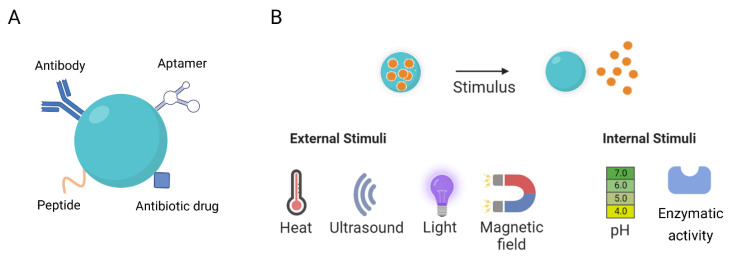
Schematic representation of (**A**) ligand-functionalized NPs and (**B**) stimuli-responsive NPs used in targeted therapy.

NPs can be targeted to infection sites passively or actively. The passive targeting of NPs relies on extended circulation and preferred accumulation in the infection site due to increased blood vessel permeability. While healthy vasculature acts as a barrier, infection-induced inflammation makes the vasculature surrounding the infection site more permeable, allowing for penetration by NPs [17]. In turn, actively targeted NPs contain ligands that bind to receptors, which are specifically expressed in bacterial cells [18]. Various ligands have been reported for bacterial targeting, including antibodies, aptamers, bacteria-binding peptides, and antibiotic drugs (Figure 1A), all of which present their own advantages and disadvantages, as detailed in Table 1. Antibodies are the most recognized ligand for targeted therapy, as they recognize specific receptors on bacterial cells. However, the high costs and difficulty of synthesizing high-quality antibodies, as well as their high molecular weight, are the main limitations to their application, highlighting the importance of developing more effective targeting strategies [19]. In recent years, other approaches, such as the use of aptamers, have been described. Aptamers are single-stranded oligonucleotides or peptides that fold into three-dimensional structures and bind to molecular targets such as cell receptors. In addition to presenting equal or similar affinity/specificity to the target receptor as antibodies, aptamers offer unique advantages, including increased stability under wide ranges of pH, temperature, and osmotic pressure; smaller sizes; easier modification and immobilization; and improved reproducibility [20]. Peptides are other suitable candidates to increase the targeting capacity of NPs to bacteria. Peptides present numerous benefits over antibodies, including decreased immunogenicity, greater penetration, cheaper production cost, and simpler synthesis and modification. However, their low target affinity (1–10% of binding affinity compared to antibodies) and metabolic instability are some of the limitations of peptides [21]. Although less explored, the use of antibiotic drugs as targeting ligands is another suitable strategy to direct antimicrobial agents to bacteria. Vancomycin is an example of a well-known antibiotic agent with targeting properties that acts by establishing hydrogen-bond interactions with the terminal D-alanyl-D-alanine moieties of bacterial cell walls. 

In addition to using ligands that specifically bind to bacterial cell receptors, stimuli-responsive nanocarriers that respond to physiological changes induced by bacteria can also be used to target infection sites. The most well-known endogenous stimuli-responsive systems for targeted MRSA therapy are pH-responsive NPs. In fact, MRSA infections are characterized by low pH at the site of infection [24]. Therefore, this feature can be used to design NPs that respond specifically to acidic environments [16]. Enzyme-responsive NPs have also been explored, as they can selectively react with enzymes specifically expressed in bacterial cells, leading to the targeted delivery of antibacterial agents. Exogenous stimuli-responsive NPs have also been proposed for the release of drugs in the desired tissue. In this approach, exogenous physical stimuli, including temperature, light, electricity, magnetic fields, or ultrasound, are applied to stimulate the release of drugs in the target tissue [25]. Figure 1B shows a schematic representation of stimuli-responsive NPs for drug delivery.

## 3. Targeted Nanoparticles for MRSA Therapy

**Figure 2:**
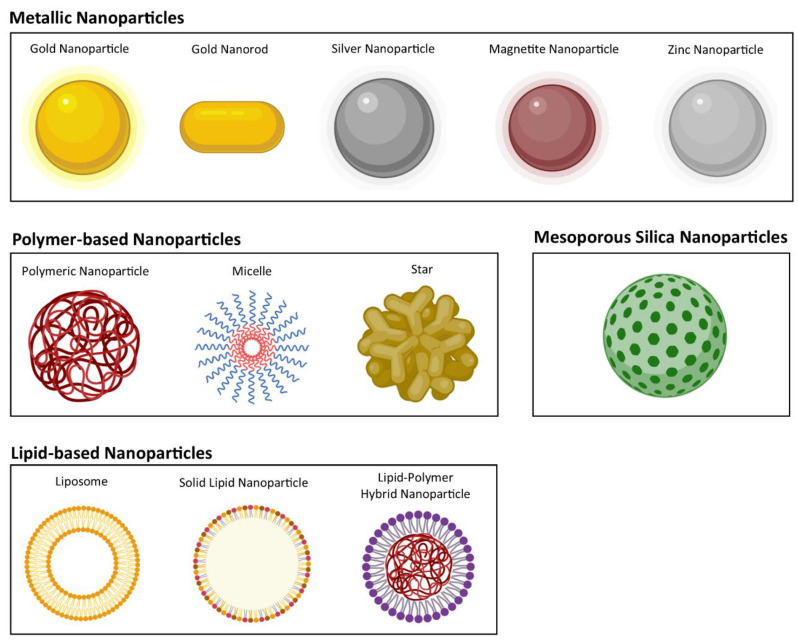
Schematic representation of the diverse types of NPs already developed for the targeted therapy of MRSA. NPs are not drawn to scale.

Over the last decade, diverse types of NPs have been proposed for the targeted therapy of MRSA, including metallic, polymer-based, mesoporous silica, and lipid-based NPs, as depicted in Figure 2. A detailed description of the NPs developed for the targeted treatment of MRSA is provided in the following sections.

### 3.1. Metallic Nanoparticles

Metallic NPs are the most commonly used type of NPs in MRSA therapy and act as both antibacterial agents and drug nanocarriers. Metallic NPs have the ability to eradicate microorganisms by disturbing their structure and functions [5]. Specifically, these NPs are capable of disrupting the bacterial cell wall and cell membrane when the positively charged ions of NPs bind to negatively charged components. This leads to the formation of pores in the membrane, which allows cytoplasmic content to leak from the bacteria, potentially leading to cell death. Moreover, the entry of NPs into the bacterial cytoplasm induces ROS formation, which may cause DNA damage and cell death [2]. Various metallic NPs have been proposed for the targeted treatment of MRSA, including gold (Au) nanostructures, silver (Ag) NPs, magnetite NPs, and zinc (Zn) NPs, which are detailed in Table 2.

#### 3.1.1. Gold Nanoparticles

Au NPs have attracted much attention for targeted therapy against MRSA as antibacterial agents. Millenbaugh et al. (2015) designed Au NPs conjugated to monoclonal antibodies specific to *S. aureus* peptidoglycan (immunoglobulin G3 isotype) and combined them with pulsed laser exposure [26]. The efficacy of the nanosystem was tested against an MRSA strain, and the results revealed that neither NP functionalization nor laser exposure alone reduced MRSA strain viability. However, combining functionalized Au NPs with pulsed laser exposure reduced MRSA survival to 58%.

Kurui et al. (2019) also evaluated the antibiotic efficacy of antibody-conjugated Au NPs combined with pulsed laser therapy against MRSA biofilms [14]. Laser irradiation alone or combined with non-conjugated Au NPs did not affect the biofilm viability. However, when combined with antibody-conjugated Au NPs, 96% of the biofilm was removed, and detachment of bacteria from the glass surface occurred, confirming the successful targeting of NPs to the biofilm. Additional data confirmed that NP conjugation with the *S. aureus* peptidoglycan monoclonal antibody led to a sevenfold increase in binding to MRSA biofilm compared to non-conjugated Au NPs. Additionally, the benefits of the developed targeted NPs followed by 24 h of treatment with the antibiotic gentamicin were evaluated. Gentamicin alone or with laser therapy reduced the biofilm viability compared to the untreated group. Combining conjugated NPs, laser therapy, and gentamicin led to a greater decrease in MRSA viability. The authors concluded that NP-targeted laser therapy potentiates gentamicin’s activity against MRSA by destructing the matrix and cellular components of the biofilm.

**Figure 3:**
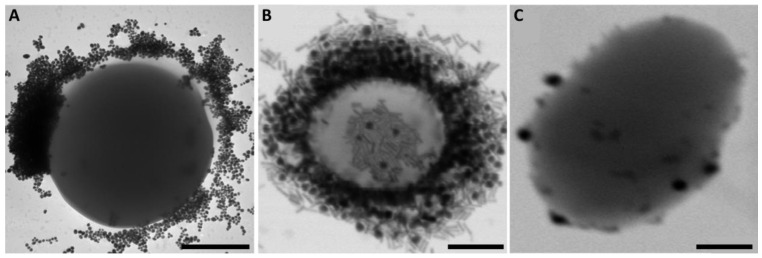
Transmission electron microscopic images of (**A**) DNA aptamer-functionalized Au NP MRSA conjugate, (**B**) DNA aptamer-functionalized Au NR MRSA conjugate, and (**C**) DNA aptamer-functionalized Au NP *E. faecalis* conjugate. Scale bars correspond to 200 nm. Figure adapted with permission from [27].

In turn, Ocsoy et al. (2017) developed DNA aptamer-functionalized Au NPs and Au nanorods (NRs) for the inactivation of MRSA with photothermal therapy [27]. A DNA aptamer was specifically selected to target the MRSA surface. Both formulations showed uniform and monodisperse populations, with diameters of 15 nm for Au NPs and mean length and width of 60 nm and 12 nm, respectively, for Au NRs. Both modified Au nanocarriers accumulated on the MRSA surface, as shown in Figure 3A,B. In contrast, the nanocarriers did not bind to *Enterococcus faecalis* cells used as negative controls, as shown in Figure 3C, with only a few NPs adsorbed on the cell surface. Although both modified Au nanocarriers bound to MRSA, only Au NRs inactivated over 95% of cells due to their higher longitudinal absorption of near-infrared (NIR) radiation and stronger photothermal conversion than that of Au NPs. 

Vancomycin-modified Au NPs were proposed by Wang et al. (2019) to kill MRSA under NIR laser irradiation, since vancomycin targets Gram-positive bacteria by binding to the D-Ala–D-Ala moiety of the cell wall [28]. The NPs presented mean sizes of 100 nm and zeta potential values of −3 mV. Scanning electron microscopic images confirmed the targeting ability of vancomycin to MRSA. While few non-modified Au NPs were observed on the MRSA surface, numerous vancomycin-modified Au NPs were noticed on the bacterial surface. Moreover, in a bacterial suspension of MRSA and *Escherichia coli*, vancomycin-modified Au NPs surrounded only MRSA cells, with no NPs found around *E. coli* cells. The authors then assessed the antibacterial efficacy and safety of NPs in an animal infection model. MRSA-infected mice were established by injecting an MRSA suspension in a 1 cm diameter wound. The application of an NP suspension in the infected wound followed by NIR irradiation improved wound healing, with no toxicity for the major organs.

Hu et al. (2017) designed pH-responsive Au NPs for the targeted therapy of MRSA under NIR light irradiation [29]. To that end, the surfaces of NPs were modified with mixed self-assembled monolayers (SAMs) consisting of strong electrolytic (10-mercaptodecyl)trimethylammonium bromide (HS-C10-N4) and weak electrolytic 11-mercaptoundecanoic acid (HS-C10-COOH). Due to the pH-responsive transition of the electrolytics from negative to positive charge, the proposed nanosystem strongly adhered to the MRSA biofilm (pH 5.5), while it dispersed in healthy tissues (pH 7.4). As a result, Au NPs exposed to NIR light decreased the number of living bacteria. In contrast, the proposed therapy did not affect the cell viability of mouse embryonic fibroblasts (3T3), proving to be safe for healthy tissues. The in vivo antibacterial activity of the NPs combined with NIR light was assessed in rabbits by injecting an Au NP suspension into a subcutaneous abscess created via local subcutaneous infection with MRSA. The treatment led to a rapid increase in MRSA biofilm temperature (to 55 °C) and bacterial death without damaging the healthy tissues around the biofilm.

The use of pH-responsive Au nanocarriers combined with photothermal therapy was also proposed by Liu et al. (2018) as a targeted MRSA therapy [30]. Polydopamine (PDA)-coated Au NRs were functionalized with glycol chitosan because of its pH sensitivity and loaded with Ag^+^ ions, as they are able to damage bacterial membranes. The population showed a mean length of 68 nm and diameters of 21 nm. In an acidic environment (pH 6.3), the NRs showed a positive zeta potential, which resulted in strong electrostatic interactions with MRSA. On the contrary, NRs exhibited a slightly negative charge at physiological pH (7.4). The results revealed that 43% of NRs bound to the MRSA surface at pH 6.3, as opposed to pH 7.4, with less than 4% adhesion to mouse embryonic fibroblasts and immortalized human keratinocytes (HaCaT cells). As a result, full bacterial inactivation was observed in vitro. The in vivo biodistribution and antibacterial activity of the developed nanosystem were assessed in a murine model of MRSA infectious abscesses established by subcutaneous injection of an MRSA suspension. The NRs were intravenously injected, and after 24 h, they were observed at the abscess site but not in the healthy skin. Consequently, treated mice showed no inflammation or abscesses on the dorsal surface, while control mice presented with abscesses and red swelling of the skin. Moreover, no damage was noticeable in the surrounding healthy tissues, nor in the heart, liver, spleen, lungs, or kidneys.

Karaagac et al. (2021) produced oleylamine (OLA)-capped Au NPs conjugated with 3-aminophenylboronic acid (3-APBA) as a targeting ligand for bacterial recognition [31]. The prepared nanosystem presented a mean size of 24 nm and a monodisperse population. The results indicated that 3-APBA binds to the NP surface via the amine group and to the glycan on the MRSA through a cis-diol configuration between the diol group of 3-APBA and glycoprotein on the MRSA membrane. However, the authors did not evaluate the antibacterial activity of the proposed NPs.

Kuo et al. (2016) proposed another strategy based on a synthetic peptide to direct Au NPs to MRSA [32]. DVFLG is a penta-peptide with a binding affinity towards *S. aureus* and MRSA that was therefore immobilized on the NP surface. The peptide was modified with arginine, which targets negatively charged bacteria, and tryptophan, which penetrates bacterial cell membranes, inducing their disruption. The functionalized Au NPs displayed a mean size of 9 nm and a zeta potential value close to −25 mV. Data demonstrated that the nanosystem slowed down the growth of MRSA in a concentration-dependent manner. Furthermore, more tryptophan molecules led to greater antibacterial activity. TEM images revealed that Au NPs anchor to the surface of MRSA. In addition to *S. aureus* and MRSA, the NPs showed binding affinity toward other pathogenic bacteria, including *Staphylococcus saprophyticus*, *Staphylococcus epidermidis*, *E. faecalis*, *Enterococcus faecium*, *VRE1*, and *VRE4*. Nevertheless, they exhibited low toxicity toward non-target cells, including macrophages and red blood cells. 

In addition to acting as antibacterial agents, Au nanostructures have also been used as nanocarriers for the targeted delivery of well-known antibiotics. Meeker et al. (2016) incorporated daptomycin into PDA-coated Au nanocages and conjugated them with a polyclonal antibody against staphylococcal protein A (aSpa) to target the cell surface of *S. aureus* including MRSA [33]. After laser irradiation, targeting specificity was confirmed by revealing that conjugated Au nanocages kill significantly more MRSA cells than unconjugated nanocarriers. Furthermore, data proved the lack of binding of conjugated Au nanocages to mammalian cells and reduced killing of *S. epidermidis*, a protein A-negative bacteria species. Later, the same group (2018) conjugated the same Au nanocages with two distinct antibodies against two different *S. aureus* lipoproteins: anti-lipoprotein antibody (aLpp) and anti-manganese transporter antibody (aMntC) [34]. These lipoproteins are overexpressed in *S. aureus* compared to planktonically grown cells. The targeting ability of both antibody-conjugated nanocarriers loaded with daptomycin compared to unconjugated daptomycin-loaded Au nanocages was confirmed, as well as their in vitro antibiotic activity against MRSA.

#### 3.1.2. Silver Nanoparticles

Ag is another effective and commonly used antibacterial agent. In addition to the above-mentioned mechanisms common to all metals, Ag suppresses cell division by interacting with DNA and RNA and interrupts signal transduction [44]. Therefore, Ag NPs have emerged as promising antibacterial agents. For instance, Xu et al. (2021) recently developed Ag NPs decorated with chlorin e6 (Ce6) and poly[4-O-(α-D-glucopyranosyl)-D-glucopyranose] (GP) for the targeted therapy of MRSA [35]. Upon laser activation, Ce6 produces ROS, which trigger the release of Ag^+^ from the Ag NP core to kill the bacteria. In turn, GP was introduced as a bacteria-specific targeting ligand, since GP recognizes bacteria through GP-mediated transporters, which are present in the bacteria surface, unlike mammalian cells. GP/Ce6 Ag NPs showed a well-defined spherical form, with average sizes of 50 nm and zeta potential values close to −10 mV. The targeting ability of NPs was validated by revealing their presence on the MRSA surface and not on the surface of mouse embryonic fibroblasts. The in vivo antibacterial effect of GP/Ce6 Ag NPs was assessed in an MRSA-infected mouse model built by injecting an MRSA suspension on their back. The results revealed a bacterial survival rate of 3%. As a result, accelerated wound repair was observed. Moreover, the authors demonstrated the excellent in vivo biocompatibility of the nanosystem.

Zuo et al. (2020) proposed enzyme-responsive Ag NP assemblies (ANAs) against MRSA [36]. ANAs were prepared from two enzyme-responsive branched copolymers: oligopoly(ethylene glycol)methyl ether methacrylate (OEGMA) and oligo-poly(ethylene glycol)methacrylate (OEGMA-OH). These structures specifically collapse when exposed to serine protease-like B enzyme proteins secreted by MRSA [36]. The mean size of ANAs was around 170 nm, while Ag NPs presented sizes between 20 and 50 nm. The in vitro MRSA killing rate of ANAs was 87%, while that of non-responsive Ag NPs was 65%, implying that ANAs exhibited enhanced antibacterial activity due to their targeting ability. Then, the authors investigated the antibacterial activity and safety of NPs in MRSA-infected rats developed by inoculating an MRSA suspension in a 1 cm diameter wound. Wound-healing experiments demonstrated that ANAs accelerated the healing of MRSA infections compared to control Ag NPs, with fewer bacterial colonies found in the ANAs-treated group. Furthermore, ANAs did not induce toxicity in the major organs.

Huo et al. (2014) prepared Au-Ag NPs for the targeted treatment of MRSA-induced pneumonia [37]. Au formed the NP core, while Ag produced a shell around the NP. The NP surface was functionalized with an anti-MRSA antibody, which selectively binds to MRSA surface. The modified NPs showed a mean diameter of 32 nm and a neutral surface charge. Compared to unconjugated Au-Ag NPs, antibody-modified NPs showed an 11-fold enhancement in targeting MRSA in vitro. The results also revealed that higher amounts of conjugated NPs are taken up by MRSA (78%) compared to bacteria without MRSA antigen, including *E. coli*, *Bacillus subtilis*, and *S. aureus* (<10%), confirming the selectivity of the nanosystem for MRSA. In vivo studies using a ventilator-associated pneumonia rat model (created by endotracheal MRSA administration) demonstrated that the conjugated NPs accumulated in MRSA-rich areas, reducing the inflammation by reducing bacterial growth and cytokine production. No long-term in vivo toxicity was noticed.

The combined use of Ag NPs with antibiotics can often result in an improvement in the overall antibacterial effects of the nanosystem. Huang et al. (2021) recently designed vancomycin-loaded Ag NPs, the surface of which was modified with a platelet membrane [8]. Platelets have the ability to recognize inflammatory cells and reduce macrophage uptake [8]. NPs showed a mean size and zeta potential of 150 nm and −25 mV, respectively, and an encapsulation efficiency (EE) of 81%. The targeted NPs presented a pH-responsive release, with vancomycin and Ag^+^ being released in higher amounts at pH 6.5 than at 7.4. The modified vancomycin-loaded Ag NPs exhibited a greater ability to inhibit MRSA growth in vitro than unmodified NPs and free vancomycin. Similar conclusions were drawn using an MRSA pneumonia mouse model established by tracheal injection of an MRSA suspension, with no apparent toxicity.

A different strategy was proposed by Chen et al. (2019) [38]. The authors created a nanostructure made of zeolitic imidazolate framework-8-polyacrylic acid (ZIF) for the delivery of ammonium methylbenzene blue (MB), a photosensitizer antibacterial agent. The structure was then coated with Ag NPs, followed by a second coating with vancomycin and polyethylene glycol (PEG) to increase the surface hydrophilicity and enhance the targeting property of the nanosystem toward Gram-positive bacteria. Vancomycin and PEG modification increased the number of NPs recognizing MRSA compared to non-modified NPs, resulting in a higher level of biofilm eradication. The biocompatibility and therapeutic efficacy of the NPs were investigated in a bacterial endophthalmitis rabbit model created through MRSA injection into the vitreous cavity of the animal’s eye. In vivo results demonstrated the biocompatibility of the NPs following their injection into animal eyes combined with laser therapy. Less inflammatory cells were detected in the NP-treated group than in the PBS and vancomycin-treated groups, validating their therapeutic efficacy in vivo.

#### 3.1.3. Magnetite Nanoparticles

Magnetite (Fe_3_O_4_) NPs have also been proposed for MRSA therapy due to their magnetic responsiveness and good targeting capacity. Wang et al. (2018) developed magnetite NPs to treat MRSA by photodynamic inactivation [39]. The NP surface was functionalized with a photosensitizer, hematoporphyrin, and a monoclonal MRSA antibody to improve the targeting capacity of the NPs. The NPs, measuring around 100 nm, were successfully captured by MRSA (96%), unlike mouse fibroblasts (L-929 cells) (6%). Consequently, after irradiation, functionalized NPs showed selective killing ability for MRSA with minimum damage to healthy fibroblasts. The authors established MRSA-infected mice by applying an MRSA suspension to skin wounds on the animals’ backs. Likewise, the MRSA infection rate of mice with skin infection decreased to 38% under light irradiation.

Zhang et al. (2020) designed chitosan-modified magnetite NPs for MRSA therapy [40]. In acidic environments, the amino groups of chitosan protonate, promoting the electrostatic binding of the NPs with the negative bacterial cell membranes. The modified NPs presented uniform particle sizes of about 100 nm and zeta potential values of 35 mV. Under white light irradiation, the system decreased the in vitro MRSA colonies by 98%.

Chen et al. (2008) proposed titania-coated Fe_3_O_4_ NPs to combat MRSA due to the antimicrobial properties of both materials [41]. Immunoglobulin G (IgG) from human serum was bound to the surface of magnetic NPs, since it recognizes several pathogenic bacteria, including MRSA, based on pseudo-immune interactions between IgG molecules and the binding proteins on the bacterial surfaces. The authors demonstrated the ability of the NPs to target MRSA; however, MRSA cell growth inhibition was not evaluated.

A different approach was recently proposed by Ocsoy et al. (2021) by forming magnetite NPs on the surface of graphene oxide (GO), forming a magnetic GO (MGO) [42]. In addition to converting NIR light to heat, GO acted as a platform for DNA aptamer conjugation. The aptamer sequence was specifically selected for active MRSA targeting. The authors demonstrated that MGO induces local heating and MRSA cell death under NIR laser irradiation. MGO modification with the DNA aptamer enhanced MRSA cell inactivation, confirming the targeting capacity of the nanosystem. 

#### 3.1.4. Zinc Nanoparticles

Zinc oxide (ZnO) has been approved by the FDA for a variety of pharmaceutical applications. ZnO NPs are particularly interesting for antimicrobial applications due to their good antibiotic properties and biocompatibility. Chen et al. (2015) designed UBI29-4 peptide-conjugated ZnO quantum dots for MRSA-targeted therapy [18]. The quantum dots showed mean diameters and zeta potential values of 20 nm and −25 mV, respectively. Ubiquicidin (UBI)_29-41_ is a cationic antimicrobial peptide that distinguishes bacterial cells from general inflammation and cancer cells in vivo. This selectivity is due to the interaction of the peptide’s cationic domains with the anionic bacterial surface. While negatively charged lipoteichoic acid and phospholipids are exposed on bacterial surfaces, they face the cytoplasm in healthy or malignant cells. Furthermore, the presence of UBI_29-41_ increased the specificity of the quantum dots to MRSA. The authors also studied the in vitro anti-MRSA activity of modified ZnO quantum dots functionalized with methicillin. The results revealed neither free methicillin nor modified ZnO quantum dots affected MRSA viability. Combining methicillin with modified NPs resulted in positive antibacterial effects.

Persistent luminescence NPs (PLNPs) composed of zinc gallogermanate were developed by Yan et al. (2021) for real-time monitoring of MRSA therapy [43]. PLNPs are promising optical materials that retain their luminescence after the excitation light has been withdrawn. The surface of the NPs was then coated with mesoporous silica to host Au NPs. Further functionalization of the NPs with chitosan-benzeneboronic acid ensures bacterial targeting by binding to peptidoglycans on the bacterial cell wall. The targeted NPs damaged the cell wall, resulting in 99% eradication of MRSA cells in vitro at pH 5.5. No antibacterial activity was detected at pH 7.0. An MRSA-infected subcutaneous abscess mouse model was established by subcutaneous injection of an MRSA suspension into the mouse’s back to evaluate the in vivo targeting ability and therapeutic activity of the NPs. The luminescence signal in the abscess region appeared 1 h after the administration of the targeted NPs. In contrast, no luminescence signal was noticed in the PBS- and non-targeted NP-treated mice. Consequently, the abscess and inflammation on the skin of the mice treated with targeted PLNPs disappeared on day 8, while remaining in the PBS and in non-targeted NP groups.

### 3.2. Polymeric Nanoparticles

Polymeric NPs are made up of polymers derived from natural or synthetic sources [13]. Like other types of NPs, polymeric NPs are promising DDSs that allow for the controlled and sustained release of drugs, which improves drug efficacy. Some polymers can also act as antibacterial agents by themselves. Numerous polymers have been employed in the production of NPs for anti-MRSA therapy, such as poly (lactic-co-glycolic acid) (PLGA) and PDA. Table 3 describes the polymeric NPs developed so far for targeted MRSA therapy. 

#### 3.2.1. PLGA Nanoparticles

PLGA is an FDA-approved synthetic copolymer with good biocompatibility and biodegradability. PLGA NPs are among the most widely researched NPs and have been used as DDS of antimicrobial agents to increase their therapeutic efficacy [4]. Chen et al. (2022) recently prepared PLGA NPs loaded with a lipophilic fluorescent dye, then coated with an M2 macrophage-derived cell membrane for treatment of MRSA infections [45]. While IR780 was used due to its photothermal conversion efficiency, M2 macrophage-derived cell membrane was added because M2 macrophages target the inflammation microenvironment and active anti-infection immune responses. The NPs exhibited a diameter of about 230 nm and a zeta potential of −8 mV. The coated NPs combined with ultrasound effectively inhibited MRSA growth in vitro. In an MRSA-infected mouse model established by intramuscular injection of a MRSA suspension, the modification of PLGA NPs with the M2 macrophage membrane enhanced the accumulation of the NPs at the site of inflammation by 2.3-fold. As a result, fewer blood flow signals, slighter muscular edema, and less clumped bacterial infiltration were observed. The DDS showed good biocompatibility in vivo.

Ucak et al. (2020) created aptamer-functionalized PLGA NPs for the targeted delivery of the antibiotic teicoplanin to MRSA [46]. The NPs presented sizes and zeta potential values of 230 nm and −30 mV, respectively, and EE of 98%. Release studies showed that most of the drug was released in the first 2 h (60%), reaching 75% in 10 days. Conducting colocalization experiments, the authors demonstrated the targeting ability of the DDS to *S. aureus* over *S. epidermidis* cells. Nevertheless, the targeting ability of aptamer-functionalized NPs was not compared to non-modified NPs. The functionalized PLGA NPs alone did not show anti-MRSA effect in vitro. However, encapsulating teicoplanin into non-functionalized NPs decreased the minimum inhibitory concentration (MIC) values of teicoplanin by twofold. By functionalizing the teicoplanin-loaded NPs with the aptamer, a 64-fold decrease in MIC was obtained compared to free teicoplanin.

#### 3.2.2. Other Polymeric Nanoparticles

Although less studied, other polymer-based NPs have been proposed for targeted MRSA treatment. Polydopamine (PDA) NPs are promising polymeric NPs for cell eradication due to their excellent photothermal conversion efficiency under external light irradiation and low cytotoxicity. Hence, Hu et al. (2019) developed vancomycin-conjugated PDA NPs for the photothermal killing of MRSA [47]. Because of multivalent hydrogen-bond interactions between vancomycin and the terminal D-alanyl-D-alanine moieties of the MRSA cell walls, vancomycin-modified NPs can specifically target MRSA rather than mammalian cells. The NPs presented average sizes and zeta potential values of 120 nm and −4 mV, respectively. The functionalization of NPs with vancomycin enhanced their adhesion to the MRSA surface. In vivo studies were conducted in mice subcutaneously injected with MRSA on their backs. After injecting an NP suspension into MRSA-infected mice via the tail vein, the NPs rapidly targeted the MRSA-infected site and adhered to the bacterial surface under NIR light. No NPs were found in any other body sites. As a result, the functionalized NPs killed MRSA, with no damage to the surrounding healthy tissues.

Kim et al. (2021) prepared 40 nm micelles made of an amphiphilic DNA block copolymer, presenting a core of hydrophobic polystyrene and a corona of densely packed DNA strands for selective capture of Gram-positive bacteria, including MRSA [48]. Fluorescence confocal images revealed that the NPs efficiently selected Gram-positive strains over Gram-negative strains. This targeting ability is due to the interaction between densely packed DNA strands and the peptidoglycan layers of Gram-positive bacteria. Magnetic NPs were then incorporated into the polymeric micelles for the magnetic capture of bacteria. The removal efficiency of Gram-positive bacteria was significantly higher than that of Gram-negative bacteria. Specifically, over 90% of MRSA strains were captured. However, the ability of the nanosystem to eradicate MRSA was not evaluated.

Sonawane et al. (2020) synthesized vancomycin-loaded micelles made of an AB2 type amphiphilic block copolymer for pH-triggered antibiotic delivery [49]. The NPs showed size, zeta potential, and EE of 130 nm, −4 mV, and 40%, respectively. The pH-dependent drug release property of the micelles was confirmed, which resulted in enhanced in vitro antibacterial activity of vancomycin against MRSA at pH 6 compared to physiological pH. The in vivo antibacterial activity of the vancomycin-loaded micelles was investigated using a skin MRSA infection mouse model created by intradermal injection of an MRSA suspension in the animals’ back. Vancomycin-loaded NPs showed superior ability in treating MRSA infections than the free drug. Blank micelles did not reveal antibacterial properties.

Guo et al. developed vancomycin-coated polypyrrole NPs for the removal of MRSA infections [50]. While polypyrrole was employed due to its significant photothermal conversion and biocompatibility, vancomycin was used as a targeting ligand. The NPs presented mean diameters and zeta potential values of 90 nm and 20 mV, respectively. The in vivo efficacy of the NPs was evaluated against MRSA-infected mice with subcutaneous abscesses created by injecting an MRSA suspension into the backs of the mice. Vancomycin-coated NPs showed a greater ability to inhibit MRSA infection than non-modified NPs. Moreover, no heat conduction in the tissue surrounding the abscess was detected, implying the safety of the NPs for the periphery healthy tissues.

Glucosamine-functionalized star polymers (20 nm) consisting of polylysine and glycopolymer arms were proposed by Wong et al. (2016) [51]. While polylysine induces bacterial death, glycopolymer was used because of its capacity to infiltrate the peptidoglycan layer found only in the bacterial cell wall. In vitro results demonstrated that the antimicrobial efficacy of the NPs was selective towards Gram-positive bacteria, including MRSA, because of the resemblance between the glucosamine moieties and the bacterial peptidoglycan layer. Additionally, the NPs exhibited good mammalian cell biocompatibility and were non-hemolytic.

### 3.3. Lipid Nanoparticles

Lipid NPs have gained popularity in the pharmaceutical industry as potential DDS for various therapeutic agents. In recent years, lipid NPs including liposomes, solid lipid NPs (SLNs), and lipid–polymer hybrid NPs (LPHNs), have been investigated for the targeted delivery of therapeutics for MRSA therapy, as listed in Table 4.

#### 3.3.1. Liposomes

Liposomes represent the earliest generation of lipid NPs. Composed of amphipathic phospholipids and shaped in spherical bilayers, liposomes present great biocompatibility. Due to their structure, liposomes can transport both hydrophobic and hydrophilic molecules, proteins, or nucleic acids [59]. Aiming to improve the efficacy of vancomycin for MRSA therapy, targeted vancomycin-loaded liposomes have been proposed by various researchers. For instance, Vanamala et al. (2021) prepared folate-decorated vancomycin-loaded liposomes after demonstrating the overexpression of folate receptors in MRSA-infected tissues compared to healthy tissues [52]. The liposomes were composed of hydrogenated soybean phosphatidylcholine (HSPC), cholesterol, and 1,2-distearoyl-sn-glycero-3-phosphoethanolamine (DSPE). The functionalized DDS presented sizes of around 150 nm, neutral zeta potential values, and controlled vancomycin release. A mouse model of thigh infection was established by injecting MRSA into the thighs of mice. Compared to free vancomycin, folate-decorated liposomes showed enhanced accumulation in MRSA-infected thigh tissues of a mouse model, resulting in a higher bactericidal effect and reduced accumulation in, healthy organs, such as kidneys and livers.

**Figure 4:**
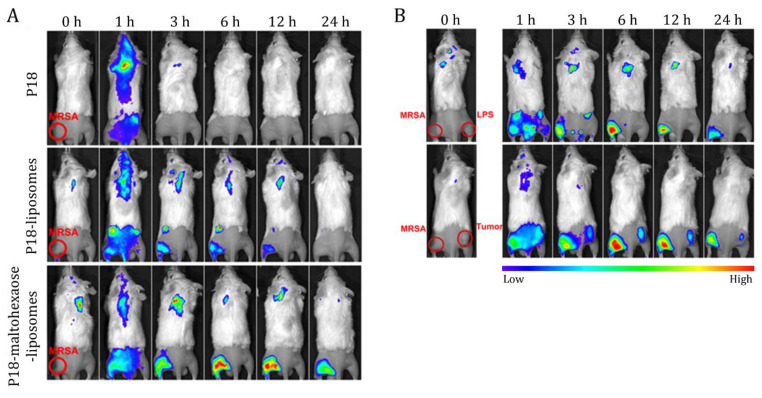
Near-infrared fluorescence images of (**A**) an MRSA-infected mouse model after tail vein injection of free P18; non-modified, P18-loaded liposomes; and maltohexaose-modified, P18-loaded liposomes and (**B**) an MRSA-infected mouse model with LPS infection or 4T1 tumor after tail vein injection of maltohexaose-modified, P18-loaded liposomes. Figure adapted with permission from [53].

A distinct approach was proposed by Pang et al. (2019), which involved encapsulating the sonosensitizer purpurin 18 (P18) into liposomes composed of 1,2-dimyristoyl-sn-glycero-3-phosphoglycerol (DMPG) and cholesterol [53]. The liposome surface was modified with maltohexaose for bacterial targeting, as it can selectively target bacteria but not mammalian cells through the bacteria-specific maltodextrin transporter pathway. The prepared DDS presented sizes of around 150 nm, a zeta potential of −27 mV, and EE of 95%. The targeting ability of the modified NPs was investigated in an MRSA-infected mouse model created by injecting an MRSA suspension into the animals’ left rear thigh muscle. As shown in Figure 4A, free P18 was distributed in all regions of the animals’ body but was rare in the MRSA-infected site. The maltohexaose-modified, P18-loaded NP group displayed increased fluorescence emission in the infection site compared to the free P18 and non-modified, P18-loaded liposome groups, which is attributed to the effective bacteria targeting of maltohexaose. The authors then studied the specificity of the maltohexaose-modified P18-loaded liposomes for MRSA in mouse models presenting either lipopolysaccharide (LPS)-induced sterile inflammation or cancer, in addition to MRSA-induced infection. As shown in Figure 4B, the NPs quickly reached the MRSA-infected site, unlike the inflammation site and mammary carcinoma cells (4T1). The biocompatibility and effectiveness of the targeted DDS in killing MRSA upon ultrasound irradiation were validated by magnetic resonance imaging using an MRSA-induced myositis mouse model.

#### 3.3.2. Solid Lipid Nanoparticles

A novel generation of lipid NPs, SLNs, has emerged to address some shortcomings of liposomes, including complex production methods and low loading capacity. In addition to exhibiting enhanced physical stability, SLNs are easily produced at a large scale without requiring organic solvents and using the cheapest materials [59]. SLNs have been mostly explored for the targeted delivery of vancomycin against MRSA. For instance, Omolo et al. (2021) designed pH-responsive SLNs composed of oleic acid and stearyl amine for the targeted delivery of vancomycin to acidic bacterial infectious sites [54]. The NPs had average sizes, zeta potential, and EE of approximately 60 nm, −6 mV, and 50%, respectively. The in vitro drug release study showed that vancomycin was released from the NPs more quickly at pH 6.0 than at pH 7.4, confirming the pH-responsiveness of the DDS due to the deportation and protonation of the complexed oleic acid and stearyl amine. The in vivo antimicrobial activity of the targeted DDS against MRSA was investigated using a skin infection mouse model obtained by intradermal injection of an MRSA suspension in the animals’ back. The results demonstrated the higher anti-MRSA activity of vancomycin-loaded SLNs compared to that of free vancomycin. While the free drug presented a reduction of 8-fold in MRSA viability, the DDS showed a 4050-fold reduction compared to the untreated group. Furthermore, SLNs did not present in vivo toxicity. 

Kalhapure et al. (2017) also proposed pH-responsive SLNs made of an acid-cleavable lipid (SA-3M) for delivery of vancomycin to acidic infection sites [24]. The diameter, zeta potential, and EE were approximately 130 nm, −26 mV, and 58%, respectively. The vancomycin release from the NPs was significantly faster at pH 6.5 than at pH 7.4, validating the pH sensitivity of SLNs. In vivo studies were conducted in an MRSA-infected mouse model established by intradermal injection of an MRSA suspension in the animals’ backs. The results revealed that vancomycin-loaded SLNs led to a 22-fold decrease in MRSA survival compared to free vancomycin. The DDS was capable of reducing histologic signs of inflammation.

Ibrahim et al. (2021) formulated the first dual pH/enzyme-responsive SLNs for targeted drug delivery [55]. The authors encapsulated vancomycin to enhance its anti-MRSA activity. SLNs were made of ascorbyl tocopherol succinate (ATS), which has dual pH/lipase-responsive properties according to the in vitro release study. Vancomycin-loaded SLNs had mean sizes, zeta potential, and EE of 107 nm, −17 mV, and 62%, respectively. The in vivo therapeutic activity of the DDS was investigated in an MRSA-infected moused model created by intradermally injecting an MRSA suspension into the back. While free vancomycin induced a 4-fold reduction in bacterial load compared to the untreated group, vancomycin-loaded NPs decreased the bacterial load by 13-fold, demonstrating that the dual-responsive SLNs improved the anti-MRSA activity of vancomycin. Additionally, vancomycin-loaded SLNs proved to be safe in vivo. 

Ghanbar et al. (2018) synthesized a new biocide named C17 (3-(4,4-Dimethyl-2,5-dioxo-imidazolidin-1-yl)-propyl]-dimethyl-tetradecyl-ammonium chloride) to be incorporated into SLNs composed of ∝-L-phosphatidylcholine [56]. The NPs were conjugated with an MRSA-specific monoclonal antibody (NYR MRSA 16) to allow for the selective delivery of C17 to MRSA. For comparison purposes, SLNs were also conjugated with a non-specific immunoglobulin G (IgG) antibody. Both formulations showed a mean size of 270 nm, a zeta potential of 5 mV, and EE close to 70%. The in vitro release of C17 from both SLNs occurred in a controlled and sustained manner. C17-loaded SLNs with specific anti-MRSA antibodies were more effective against MRSA than unconjugated or IgG-conjugated C17-SLNs. Furthermore, anti-MRSA antibody-conjugated C17-SLNs showed selective toxicity towards MRSA when incubated in a coculture of MRSA/fibroblast, MRSA/*Pseudomonas aeruginosa*, or MRSA/*E. Coli*.

#### 3.3.3. Lipid–Polymer Hybrid Nanoparticles

LPHNs are the most recent generation of DDSs to emerge in the nanomedicine field. These NPs possess a polymeric core capable of encapsulating both hydrophilic and hydrophobic drugs, surrounded by a lipid layer, combining the advantages of polymeric NPs and liposomes [60]. Hence, some LPHNs have already been developed for targeted MRSA therapy. For instance, Maji et al. (2019) proposed pH-responsive LPHNs made of OLA and polyamidoamine dendrimers for the targeted delivery of vancomycin to the infection site [16]. The NPs had sizes of around 120 nm, a zeta potential of −7 mV, and EE of 83%. In vitro release studies confirmed the pH-responsiveness of the NPs, with the drug being released faster at pH 6.0 than at 7.4 due to the protonation of the secondary amine of OLA in acidic pH, leading to an increase in the surface charge of the NPs. The antimicrobial activity of the LPHNs was investigated by infecting immortalized human embryonic kidney cells (HEK 293) with MRSA. While free vancomycin showed no antimicrobial activity, vancomycin-loaded LPHNs reduced the MRSA load. Moreover, the DDS showed no toxicity to healthy cells.

Vancomycin-loaded LPHNs with pH-responsive properties were also proposed by Makhathini et al. (2020) [57]. The authors prepared micelles consisting of an oleic acid tail and propionic acid dendrimers for the targeted delivery of vancomycin at acidic bacterial infection sites. The NPs presented sizes of 84 nm, zeta potential of 43 mV, and EE of 79%. The faster release of vancomycin at pH 6.0 than at 7.4 validated the pH-responsiveness of the NPs. This behavior was ascribed to the protonation of the tertiary amine of oleic acid-derived lipid dendritic amphiphile, which contributes to vancomycin release due to the rearrangement or disassembly of the NPs. An in vitro MRSA viability assay indicated that vancomycin-loaded LPHNs eradicated 93% of MRSA, in contrast to 58% eradicated by free vancomycin. Likewise, the DDS resulted in an eightfold reduction in the MRSA burden of infected mice (intradermal injection of an MRSA suspension) compared to the free drug.

Acknowledging the benefits of the codelivery of therapeutics, Jaglal et al. (2021) designed pH-responsive LPHNs made of oleic acid and polyallylamine for codelivery of vancomycin and 18β-glycyrrhetinic acid for MRSA treatment [58]. 18β-glycyrrhetinic acid is a natural compound with antibacterial properties extracted from *Glycyrrhiza glabra*. The optimized NPs had diameters, zeta potentials, and EE of 200 nm, −4 mV, and 70%, respectively. The in vitro release of vancomycin showed a faster pattern from LPHNs at pH 6.0 than at 7.4, resulting in higher anti-MRSA activity in the acidic environment. The pH-responsiveness of the LPHNs resulted from the protonation of oleic acid and polyallylamine at pH 6.0, which enhanced drug release due to the NPs burst. Furthermore, the DDS presented synergistic properties in terms of elimination of MRSA cells and MRSA biofilm compared to free vancomycin and 18β-glycyrrhetinic acid. In turn, bare LPHNs did not present activity against MRSA, regardless of the pH. In vitro hemolysis and biocompatibility studies conducted in human intestinal epithelial cancer cells (CACO-2) and human liver adenocarcinoma cells (HepG2) confirmed the safety of DDS.

### 3.4. Mesoporous Silica Nanoparticles

Mesoporous silica NPs (MSNs) have attracted much interest as nanocarriers for delivery of therapeutic drugs. Their unique mesoporous structure allows drugs to be loaded in the NPs’ core or surface via electrostatic adsorption, hydrophobic interactions, or covalent binding [61]. Various MSNs have been proposed for targeted MRSA therapy, as detailed in Table 5. Specifically, most MSNs have been designed to incorporate well-known antibiotics, such as vancomycin and rifampin. 

For instance, Nie et al. (2022) recently developed bone-and-bacteria dual-targeted MSNs for the targeted delivery of vancomycin to MRSA-infected bone sites [62]. D6 peptide was used to target bones because of the affinity of aspartic acid to bone tissues, while UBI_29-41_ peptide was employed as a bacteria-targeted agent because of its six positively charged residues, which interact with negatively charged *S. aureus* cell walls. The dual-targeted MSNs presented sizes, zeta potential, and EE values of 100 nm, 27 mV, and 20%, respectively. The targeting ability of the modified MSNs toward MRSA and hydroxyapatite (major natural inorganic mineral component of human bones) was demonstrated in vitro. The authors then investigated the therapeutic efficacy of the dual-targeted DDS in a rat model with an orthopedic implant infected with MRSA. The animal model was established by contaminating the implant with an MRSA suspension and implanting it in the animals’ left femurs via the femoral condyles. Treated groups included free vancomycin, vancomycin-loaded MSNs, vancomycin-loaded D6-MSNs, vancomycin-loaded UBI-MSNs, and vancomycin-loaded dual-targeted MSNs. Bone destruction around the implant was observed in the untreated group. Among all groups, vancomycin-loaded dual-targeted NPs showed the largest decrease in bone destruction and preserved bone integrity. Furthermore, the dual-targeted DDS showed high biocompatibility.

In turn, Ding et al. (2018) endowed vancomycin-loaded MSNs with targeting properties through anti-MRSA monoclonal antibody conjugation [63]. The functionalized NPs presented a mean size of 150 nm and a neutral surface charge. Vancomycin was released from the NPs in a controlled and sustained manner. The in vitro selectivity of the targeted NPs was investigated for different pathogens, including MRSA, *S. aureus*, *E. coli*, and *Pseudomonas aeruginosa*. Modified MSNs exhibited a sevenfold higher binding efficacy against MRSA than non-modified NPs. Moreover, no binding was detected in the other bacteria (*S. aureus*, *E. coli*, and *P. aeruginosa*), regardless of the antibody surface modification, validating the selectivity of modified MSNs for MRSA. Consequently, targeted NPs presented a higher antiproliferation effect of MRSA than non-targeted MSNs. Likewise, none of the formulations inhibited the proliferation of *S. aureus*, *E. coli*, and *P. aeruginosa*. Using rats infected with MRSA pneumonia established by endotracheal administration of MRSA, the authors demonstrated that antibody-conjugated, vancomycin-loaded MSNs specifically targeted the MRSA-induced lesions, reduced MRSA-induced inflammation, and were safe.

Functionalized MSNs were also proposed by Fulaz et al. (2020) for the targeted delivery of vancomycin against MRSA biofilms [64]. NP entrapment in MRSA biofilm was tested with different surface modifications, including benzene-functionalized (aromatic) and positively charged MSNs (MSNs-A), amino-functionalized and positively charged MSNs (MSNs-D), and carboxy-functionalized and negatively charged MSNs (MSNs-C). Mean diameters and EE values of the distinct formulations varied between 30 and 40 nm and 13–47%, respectively. Although negatively charged NPs (MSNs-C) showed higher EE, positively charged NPs (MSNs-A and MSNs-D) were more efficiently bound to the MRSA surface, given the negative charge of the components of the biofilm matrix. As a result, positively charged MSNs were more active in reducing biofilm cell viability.

With the aim of targeted delivery of another well-established antibiotic, Qu et al. (2022) recently fabricated rifampin-loaded MSNs (130 nm) against MRSA-associated infections [65]. Phosphatidylglycerol and phosphatidylcholine were used to modify the surface of NPs to actively recognize phospholipase-positive bacteria. The antibacterial efficacy of the DDS was investigated in a rat model of MRSA-infected wounds. In addition to being biocompatible, the nanosystem showed high efficacy against MRSA-associated infections, eliminating more MRSA compared to the rifampin-loaded, non-modified NPs and PBS groups. Rifampin-loaded modified MSNs significantly increased the epithelial gap thickness to promote wound re-epithelialization, which resulted in accelerated wound healing.

In addition to delivering well-known antibiotics, MSNs have also been used to deliver other agents with anti-MRSA properties. For instance, Xu et al. (2021) proposed chitosan and PDA-coated copper (Cu)-doped MSNs to eliminate MRSA [66]. Cu^+2^ has been reported to interact with negatively charged cell membranes, causing their destruction. PDA, together with Cu^+2^, provides photothermal properties to NPs. Glycol chitosan, a pH-sensitive chitosan derivative, was added to the formulation to improve bacteria targeting, since glycol chitosan has a positive charge in acidic environments, allowing for its adherence on negatively charged bacteria surfaces. The in vivo bacteria-targeting ability of the proposed nanosystem was assessed in a mouse model of an MRSA-infected abscesses created by subcutaneous injection of an MRSA suspension into the animals’ right flanks. The presence of glycol chitosan improved the accumulation of NPs on the bacterial abscesses, resulting in a significantly lower survival rate of MRSA (34%) compared to non-targeted NPs (75%). Targeted Cu-containing MSNs also showed improved wound-healing activity and biocompatibility.

A different strategy was proposed by Devlin et al. (2021) by preparing MSNs of around 40 nm, which were functionalized with three enzymes to target and destroy MRSA biofilms [67]. In addition to presenting anti-staphylococcal properties, lysostaphin was also used as a targeting ligand, as it specifically targets *S. aureus* bacteria, causing their lysis. Serrapeptase and DNase I were used to degrade protein and eDNA in the extracellular polymeric substance matrix, thereby enhancing biofilm eradication. Non-functionalized NPs exhibited no inherent antibacterial activity in vitro. The three-enzyme-functionalized MSNs showed a greater ability to reduce the viability of MRSA biofilms in vitro compared to single-enzyme-functionalized MSNs.

## 4. Discussion and Concluding Remarks

Over the course of more than half a century, antibiotics have saved millions of lives from various infectious illnesses. However, their overuse has led to increased bacterial resistance, posing a serious challenge in the battle against infectious diseases [68]. The use of NPs as antimicrobial agents and/or nanocarriers for antibiotics represents a promising strategy for the development of effective treatments against various antibiotic-resistant pathogens, including MRSA. However, the effective antimicrobial activity of NPs is highly dependent on their ability to reach the infection site and distinguish pathogenic bacteria from healthy cells [69].

**Figure 5:**
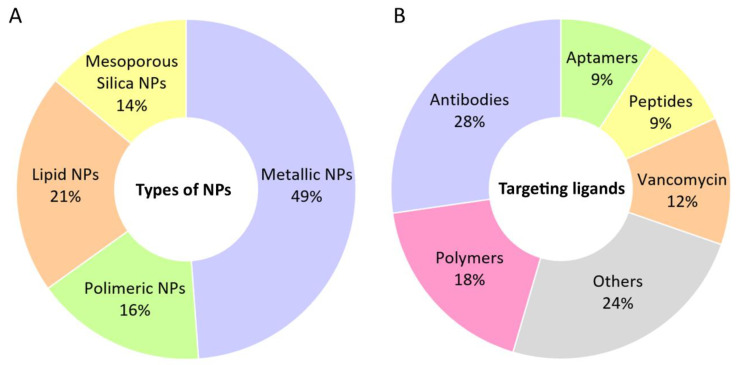
Graphical representation of the distribution of the types of (**A**) NPs developed for the targeted therapy of MRSA and (**B**) targeting ligands used for that purpose. This chart was generated based on the works included in this review.

Currently, research on NPs for targeted MRSA treatment is expanding, demonstrating some promising results and introducing new approaches to overcome AMR. Several types of NPs have been proposed, presenting different benefits and limitations. An overall examination of the studies described in this review reveals that metallic NPs account for almost half of the studies included in this article (Figure 5A). Metallic NPs are recognized for their strong antibacterial activity, enabling the resistance defenses of MRSA to be overcome without the need for antibiotics. Their ability to produce ROS and interact with the MRSA cell membrane and structures are the key mechanisms of their antibacterial effects. However, metallic NPs are not selective for bacterial cells, causing toxicity towards healthy animal/human cells, which restricts their clinical applications [70]. The use of targeting strategies is therefore mandatory to overcome this limitation. Most studies reported in this review have demonstrated the safety of metallic NPs in combination with targeting approaches.

Considerable progress has been made in research on the use of other types of NPs, such as polymeric, lipid, and mesoporous silica NPs, as DDSs for many applications. However, evidence is still scarce for targeted MRSA treatment (Figure 5A). Globally, liposomes are the type of NPs that have received the most approval for different types of clinical applications [71]. However, they remain sparingly investigated for targeted MRSA therapy, with only two studies reported to date (5%). Therefore, further investigation is needed to provide data on their efficacy and safety for MRSA therapy. 

As evidenced throughout this article, different ligands have been proposed to direct NPs to the target site. As illustrated in Figure 5B, the functionalization of the surface of NPs with antibodies is the most investigated targeting strategy, accounting for 28% of the used ligands. The interest in antibodies as targeting ligands is mainly related to their high specificity, which allows a small number of antibodies to ensure the targeting of NPs [19]. Most of the antibodies described herein are monoclonal antibodies, which offer several benefits compared to polyclonal antibodies, including higher homogeneity and specificity to a single epitope and a lower degree of cross reactivity [72]. Various groups have also referred to the use of polymers as targeting ligand for MRSA binding (18%), such as glycol chitosan, a pH-responsive polymer that exhibits a positive charge in acidic environments, allowing for its adherence to negatively charged cells wall/membranes of bacteria through electrostatic interactions. Although vancomycin, peptides, and aptamers have several advantages compared to antibodies, such as a longer shelf life, smaller size, higher stability, and lower cost, they are still the less explored ligands for this application (Figure 5B).

Moreover, various authors have developed pH-responsive NPs to direct them to the infection site, since MRSA, like many bacteria, produces acids at the infection site. This approach allows therapeutic molecules to be released in acidic environments while maintaining physiological pH [73]. This approach corresponds to 21% of the studies included in this review. However, it is important to mention that not only bacteria produce acidic environments. For example, cancer cells are known to acidify their surroundings [74]. Therefore, in addition to targeting NPs to acidic settings, it is crucial to specifically direct them to MRSA-infected sites. However, none of the reports described herein address this concern.

Interestingly, many studies (37%) have combined ligand-functionalized NPs or pH-responsive NPs with external stimuli, such as light, thermal stimulus, and magnetic field. External stimuli can be easily regulated and modified to meet the needs of each individual. Externally stimulated NPs offer improved site-specific targeting, as well as quick payload release. However, this approach demands specialized equipment and methods to generate precise stimulations [75]. Furthermore, the efficacy of this strategy depends on the tissue depth to be reached; while this approach can be useful for wound infections, it can be challenging for other infections, such as gastrointestinal infections.

The research focusing on fighting MRSA with targeted NPs is still recent, with almost 80% of the studies included in the review published in the last 5 years. However, 60% of the publications have already expanded to the in vivo phase using MRSA-infected animal models. The in vivo studies described herein validated the antimicrobial efficacy of NPS against MRSA, presenting good blood compatibility and reduced toxicity. However, few works have evaluated antimicrobial activity in MRSA biofilms, which is extremely important from a clinical standpoint. The formation of biofilms acts as a barrier to bacterial treatment [76], so it is also essential to assess the anti-biofilm activity of NPs in the future. Furthermore, none of the mentioned studies looked into intracellular MRSA infections. In recent years, it has become clear that *S. aureus* has an important intracellular component to its infection cycle, leading to infections that are difficult to treat [77]. *S. aureus* has been shown to use liver Kupffer cells (KCs) upon bloodstream infection in vivo to hide from immune cells and antibiotics [78]. In the last decade, efforts have been made to identify treatment options capable of targeting intracellular *S. aureus*. For instance, Surewaard et al. (2016) designed vancomycin-loaded liposomes, which were efficiently taken up by KCs and reduced intracellular MRSA and mortality [78]. In turn, Lehar et al. (2015) conjugated an anti-*S. aureus* antibody to the antibiotic rifalogue to specifically eliminate *S. aureus* inside mammalian cells [79]. The development of antibiotic strategies that specifically target the intracellular *S. aureus* reservoir was recently reviewed by Hommes et al. (2022) [77].

Despite the encouraging outcomes acquired from preclinical studies, research progress is not yet sufficient to translate into clinical approval, with no NPs (with or without targeting strategies) currently on the market for MRSA treatment or the treatment of any other bacterial infection. Only one clinical trial using NPs (without targeting) has been conducted for MRSA management (NCT04431440) [80], and no others are currently in progress. The authors investigated the bactericidal effect of Ag NPs against MRSA isolated from blood samples of 83 critically ill patients. The antimicrobial efficacy of Ag NPs was confirmed using the agar well diffusion method. However, it is important to highlight that the NPs were not administered to patients; therefore, care must be taken when interpreting this study.

Despite the valuable findings reviewed here, further experiments are needed to validate the benefits of targeted NPs for MRSA treatment and increase the number of successful and reliable NPs in clinical trials. The lack of clinical trials exploring the safety and efficacy of targeted NPs for MRSA is mainly due to the high costs of their development and production, so addition methods of commercial-scale production must be explored to increase the number of NPs reaching clinical trials [81]. We hope that this research can result in a nanoformulation capable of combating MRSA and, ultimately, saving lives.

## Figures and Tables

**Table 1:** Main advantages and disadvantages of the most used targeting ligands.

Ligand	Advantages	Disadvantages	Ref.
Antibodies	High specificity, long half-life, easily mass-produced	Difficulty of synthesizing high-quality antibodies, high cost, high molecular weight	[19]
Aptamers	High affinity/specificity, stability, and reproducibility; small size; easy modification and immobilization	Degradation in biological media, cross reactivity	[22]
Peptides	Higher cost-effectiveness than antibodies, small size, high binding efficiency, low metabolic consequences, decreased immunogenicity, easily mass-produced	Low target affinity, high clearance, poor pharmacokinetics, metabolic instability	[21]
Antibiotic drugs	Low molecular weight, simplicity of their conjugation, easily produced	Weak interaction with their target	[23]

**Table 2:** Metallic NPs developed for the targeted therapy of MRSA.

Type of NP	Targeting Ligand	Coating	Therapeutic Agent	Main Conclusions	Development Phase	Ref.
Au NPs	Anti-*S. aureus* peptidoglycan antibody	n.a.	n.a.	MRSA survival decreased to 58%	In vitro	[26]
Au NPs	Anti-*S. aureus* peptidoglycan antibody	n.a.	n.a.	96% of the MRSA biofilm was removed; NPs conjugation increased MRSA biofilm binding by 7-fold compared to non-conjugated NPs	In vitro	[14]
Au NPs and NRs	DNA aptamer	n.a.	n.a.	Both Au nanocarriers accumulated in the MRSA surface and not in the surface of control bacteria; Au NRs inactivated over 95% of cells; no effect for Au NPs	In vitro	[27]
Au NPs	Vancomycin	n.a.	n.a.	Vancomycin-NPs showed targeting ability towards MRSA; NPs improved wound healing in vivo; biocompatible	In vivo	[28]
Au NPs	(10-mercaptodecyl)trimethylammonium bromide and 11-mercaptoundecanoic acid	n.a.	n.a.	NPs decreased the number of living bacteria without damaging the healthy tissues around the biofilm	In vivo	[29]
Au NRs	Glycol chitosan	PDA	n.a.	NRs were observed at the inflammatory site but not in the normal skin; treated mice showed no inflammation or abscess; no damage in the surrounding healthy tissues	In vivo	[30]
Au NPs	3-APBA	OLA	n.a.	3-APBA binds specifically to the MRSA membrane	In vitro	[31]
Au NPs	DVFLG peptide modified with arginine and tryptophan	n.a.	n.a.	NPs slowed down the growth of MRSA in a concentration-dependent manner; low toxicity toward non-target cells	In vitro	[32]
Au nanocages	Staphylococcal protein A antibody	PDA	Daptomycin	Conjugated nanocages killed significantly more MRSA than unconjugated nanocages; lack of binding of conjugated nanocages to mammalian cells and *S. epidermidis*	In vitro	[33]
Au nanocages	aLpp and aMntC antibodies	PDA	Daptomycin	Targeting ability of antibody-conjugated nanocarriers compared to unconjugated nanocages; in vitro antibiotic activity against MRSA	In vitro	[34]
Ag NPs	Poly[4-O-(α-D-glucopyranosyl)-D-glucopyranose]	n.a.	Chlorin e6	Bacteria survival of 3% in MRSA-infected mice, resulting in accelerated wound repair; biocompatible	In vivo	[35]
Ag NPs	Enzyme-responsive branch polymers	n.a.	n.a.	Enhanced MRSA killing rate of ANAs, resulting in accelerated healing of MRSA infections; biocompatible	In vivo	[36]
Au–Ag NPs	Anti-MRSA antibody	n.a.	n.a.	Compared to unconjugated NPs, antibody-modified NPs showed an 11-fold enhancement in targeting MRSA in vitro; reduction in the inflammation in vivo; biocompatible	In vivo	[37]
Ag NPs	Platelet membrane	n.a.	Vancomycin	Vancomycin-loaded modified NPs exhibited a greater ability to inhibit MRSA growth than unmodified loaded NPs and free vancomycin; biocompatible	In vivo	[8]
Ag NPs	Vancomycin	n.a.	Ammonium methylbenzene blue	Increased biofilm eradication after NP functionalization; biocompatible	In vivo	[38]
Magnetite NPs	MRSA antibody	n.a.	n.a.	NPs showed selective killing ability for MRSA with minimum damage to mouse fibroblast cells; MRSA infection rate of mice with skin infection decreased to 38%	In vivo	[39]
Magnetite NPs	Chitosan	n.a.	n.a.	Decrease in MRSA colonies by 98%	In vitro	[40]
Magnetite NPs	IgG antibody	Titania	n.a.	Ability to target MRSA	In vitro	[41]
Magnetite NPs	DNA aptamer	n.a.	n.a.	Targeted NPs exhibited higher cell inactivation activity compared to non-targeted NPs	In vitro	[42]
ZnO quantum dots	UBI_29-41_ peptide	n.a.	Methicillin	UBI29-4 improved MRSA specificity; combining methicillin and modified NPs improved their individual anti-MRSA properties	In vivo	[18]
Zinc gallogermanate NPs	Chitosan-benzeneboronic acid	Mesoporous silica	n.a.	Surface modification allowed for the presence of the NPs in the inflammatory region; abscesses and inflammation on the skin of mice treated with targeted NPs disappeared but remained in the non-targeted NP group	In vivo	[43]

n.a.: not applicable. 3-APBA: 3-aminophenylboronic acid; Au NPs: gold nanoparticles; Au NRs: gold nanorods; IgG: immunoglobulin G; MRSA: methicillin-resistant *Staphylococcus aureus*; NPs: nanoparticles; OLA: oleylamine; PDA: polydopamine.

**Table 3:** Polymeric NPs developed for the targeted therapy of MRSA.

NP Composition	Targeting Ligand	Therapeutic Agent	Main Conclusions	Development Phase	Ref.
PLGA NPs	M2 macrophage membrane	IR780	The coating increased NP accumulation at the infection site, improving the antibacterial efficacy of the DDS; biocompatible	In vivo	[45]
PLGA NPs	Aptamer	Teicoplanin	Targeting capacity of the DDS to *S. aureus* over *S. epidermidis* cells; functionalization led to a 32-fold decrease in MIC compared to non-functionalized NPs	In vitro	[46]
PDA NPs	Vancomycin	n.a.	Vancomycin enhanced NP adhesion to the MRSA surface; NPs rapidly targeted the MRSA-infected site; biocompatible	In vivo	[47]
Polystyrene- DNA strand micelles	n.a.	n.a.	NPs efficiently selected Gram-positive strains over Gram-negative strains; over 90% of MRSA strains were captured	In vitro	[48]
AB2-type amphiphilic micelles	n.a.	Vancomycin	pH-dependent drug release resulted in enhanced in vitro antibacterial activity of vancomycin at basic pH; vancomycin-loaded NPs showed superior ability to treat MRSA infections relative to free drug	In vivo	[49]
Polypyrrole NPs	Vancomycin	n.a.	Modified NPs showed a higher ability to inhibit MRSA infection than non-modified NPs; biocompatible	In vivo	[50]
Polylysine glycopolymer stars	Glucosamine	n.a.	The antimicrobial efficacy of NPs was selective toward Gram-positive bacteria, including MRSA; biocompatible	In vitro	[51]

n.a.: not applicable; DDS: drug delivery system; MIC: minimum inhibitory concentration; MRSA: methicillin-resistant *Staphylococcus aureus*; NPs: nanoparticles; PDA: polydopamine; PLGA: poly (lactic-co-glycolic acid).

**Table 4:** Lipid NPs developed for the targeted therapy of MRSA.

Type of NP	Targeting Ligand	Therapeutic Agent	Main Conclusions	Development Phase	Ref.
Liposomes	Folate	Vancomycin	Compared to free vancomycin, folate-decorated NPs showed enhanced accumulation in MRSA-infected tissues, resulting in a higher bactericidal effect and reduced accumulation in kidneys and liver	In vivo	[52]
Liposomes	Maltohexaose	Purpurin 18	Modified NPs targeted the infection site; specificity for MRSA-infected sites and not inflammation sites and cancer; effective MRSA killing; biocompatible	In vivo	[53]
SLNs	Oleic acid and stearyl amine	Vancomycin	Higher anti-MRSA activity of vancomycin-loaded SLNs compared to free vancomycin	In vivo	[54]
SLNs	SA-3M	Vancomycin	Vancomycin-loaded SLNs led to a 22-fold decrease in MRSA survival compared to free vancomycin	In vivo	[24]
SLNs	Ascorbyl tocopherol succinate	Vancomycin	Free vancomycin resulted in a 4-fold reduction in bacterial load of MRSA-infected mice compared to the untreated group, while vancomycin-loaded NPs decreased the bacterial load by 13-fold; biocompatible	In vivo	[55]
SLNs	Anti-MRSA antibody (NYR MRSA 16)	C17	SLNs with anti-MRSA antibodies were more effective against MRSA than unconjugated or IgG-conjugated SLNs; selective toxicity toward MRSA	In vitro	[56]
LPHNs	n.a.	Vancomycin	Free vancomycin showed no antimicrobial activity, while vancomycin-loaded LPHNs reduced the MRSA load	In vitro	[16]
LPHNs	n.a.	Vancomycin	DDS resulted in an eightfold reduction in the MRSA burden of infected mice compared to the free drug	In vivo	[57]
LPHNs	n.a.	Vancomycin + 18β-glycyrrhetinic acid	NPs presented a synergistic effect in terms of elimination of MRSA cells and MRSA biofilm compared to free vancomycin and 18β-glycyrrhetinic acid	In vitro	[58]

n.a.: not applicable; DDS: drug delivery system; LPHNs: lipid–polymer hybrid nanoparticles; MRSA: methicillin-resistant *Staphylococcus aureus*; NPs: nanoparticles; SLNs: solid lipid nanoparticles.

**Table 5:** MSNs developed for the targeted therapy of MRSA.

Therapeutic Agent	Targeting Ligand	Main Conclusions	Development Phase	Ref.
Vancomycin	D6 and UBI_29-41_ peptides	Vancomycin-loaded, dual-targeted NPs showed the largest decrease in bone destruction and preserved bone integrity in vivo; biocompatible	In vivo	[62]
Vancomycin	Anti-MRSA antibody	Modified MSNs exhibited a sevenfold higher binding efficacy against MRSA than non-modified NPs, resulting in a higher antiproliferation effect; biocompatible	In vivo	[63]
Vancomycin	Amine, carboxyl, and aromatic groups	Positively charged NPs were more efficiently bonded to the MRSA surface, resulting in a higher capacity to reduce biofilm viability	In vitro	[64]
Rifampin	Phosphatidylglycerol and phosphatidylcholine	NP modification increased MRSA eradication compared to non-modified NPs, accelerating wound healing; biocompatible	In vivo	[65]
Copper	Glycol chitosan	NP functionalization enhanced their accumulation on the infection site; improved wound-healing activity; biocompatible	In vivo	[66]
Serrapeptase and DNase I	Lysostaphin	Functionalized NPs showed a greater ability to reduce the viability of MRSA than non-functionalized MSNs	In vitro	[67]

MRSA: methicillin-resistant *Staphylococcus aureus*; MSNs: Mesoporous silica nanoparticles; NPs: nanoparticles.

## Data Availability

The systematic literature search was conducted until November 2022 using Science Direct, Google Scholar, Scopus, and Web of Science as online databases. Key terms for the literature search included “nanoparticle”, “target”, “targeting”, “functionalized”, “Methicillin-resistant Staphylococcus aureus”, and “MRSA”. Only research papers written in English were included in this review, with no publication date limitation.
